# Effects and safety of intranasal phototherapy for allergic rhinitis: Study protocol for a single-center, randomized, double-blind, parallel, placebo-controlled, investigator-initiated, pilot study

**DOI:** 10.1097/MD.0000000000020835

**Published:** 2020-07-24

**Authors:** Jeongin Kang, Goeun Lee, Jeonghun Kim, Youngeun Kim, Sunju Park, Donghyo Lee

**Affiliations:** a Department of Ophthalmology, Otolaryngology, and Dermatology, College of Korean Medicine, Woo-Suk University, Jeonju; b Department of Oriental Rehabilitation, National Rehabilitation Center, Seoul; c Evidence-Based Healthcare Research Collaborating Center, Woo-Suk University, Jeonju; d Future Medicine Division, Korea Institute of Oriental Medicine; e Department of Preventive Medicine, College of Korean Medicine, Daejeon University, Daejeon, Korea.

**Keywords:** allergic rhinitis, intranasal phototherapy, pilot study, randomized controlled trial, study protocol

## Abstract

**Introduction:**

Allergic rhinitis (AR) is an immunoglobulin E (Ig E)-mediated inflammatory disease. Intranasal phototherapy is a promising treatment modality because it has a profound immunosuppressive effect, but the available evidence of its use for AR is insufficient. Therefore, rigorously designed randomized controlled trials (RCTs) are needed. Our objective is to describe the protocol for a feasibility trial to assess the effects and safety of intranasal phototherapy for the treatment of AR.

**Methods and analysis:**

This is a study protocol for a single-center, randomized, double-blind, parallel, placebo-controlled, investigator-initiated pilot study. A total of 40 patients with AR will be randomly assigned to the medical device or sham device group in a 1:1 ratio. The participants will receive intranasal phototherapy with a medical or sham device for 20 min 5 times a week for 2 weeks. The primary outcome will be the mean change in the Total Nasal Symptom Score (TNSS) from baseline to 2 weeks. The secondary outcomes will include the Rhinoconjunctivitis Quality of Life Questionnaire (RQLQ) score, Nasal Endoscopy Index, total serum Ig E level, and eosinophil count.

**Discussion:**

The findings of this study will provide the basis for subsequent large-scale definitive RCTs to confirm the effects and safety of intranasal phototherapy for the treatment of nasal symptoms in patients with AR who do not respond well to conventional therapy. This study may assist in the development of noninvasive treatment for patients with AR.

**Trial registration:**

This study was registered at the Korean National Clinical Trial Registry, Clinical Research Information Service (KCT0003253).

## 1 Introduction

Allergic rhinitis (AR) is an inflammatory disease of the nasal membranes that results from an immunoglobulin E (Ig E)-mediated immune reaction to allergen exposure.^[1]^ The major symptoms of AR are nasal congestion, rhinorrhea, nasal itching, and sneezing.

AR is a common respiratory disease that affects 10% to 30% of the global population.^[2,3]^ AR affects 10% of the pediatric population and 10% to 15% of the adolescent population in Korea, and it has been reported that the prevalence of persistent AR has increased over the last decade.^[4]^

AR might not be associated with severe morbidity and mortality. However, it has been reported that patients with AR often have poor quality of life (QOL). AR disrupts sleeping, interrupts learning, and reduces productivity at work.^[5]^ Moreover, AR is associated with asthma, rhinosinusitis, conjunctivitis, and chronic cough.^[6]^

For the management of AR, allergen avoidance and medication such as antihistamines, nasal decongestants, and corticosteroids are recommended as conventional therapies.^[7]^ However, avoiding allergens completely is almost impossible for most people.^[8]^ Also, there are risks of adverse effects with long-term use of medications such as rhinitis medicamentosa.^[9]^ Additionally, there are some patients whose symptoms do not improve completely even with these medications and who have some limitations and special considerations for using these medications, such as patients who are pregnant or breastfeeding.^[10]^

For these reasons, the use of less conventional treatments is particularly prevalent in patients with AR.^[11,12]^ Additionally, it was reported that more than 50% of patients with AR used multiple therapies.^[13]^ Recent studies are underway to develop new treatment options for AR patients.

As phototherapy might reduce the number of eosinophils,^[14]^ induce apoptosis of immune cells and inhibit the reaction of inflammatory mediators,^[15]^ it has been suggested that intranasal phototherapy might be effective in relieving the symptoms of AR.^[16,17]^

However, there is one comparative pilot study in Korea, and it did not include a control group.^[18]^ Thus, it is necessary to perform studies to establish evidence and assess the clinical applicability of intranasal phototherapy.

We designed a single-center, randomized, double-blind, parallel, placebo-controlled, investigator-initiated, pilot study to validate the effects and safety of intranasal phototherapy for AR.

## 2 Methods

### 2.1 Objective

The aim of this study is to describe the protocol for a randomized controlled trial designed to clinically assess the effects and safety of intranasal phototherapy for AR in comparison with those of a sham device.

### 2.2 Study design and setting

This is a double-blind, randomized, placebo-controlled, parallel-group, single-center, investigator-initiated, and pilot study. This study will be conducted at the Woosuk Korean Medicine Medical Center, Jeonju, Republic of Korea.

**Figure 1:**
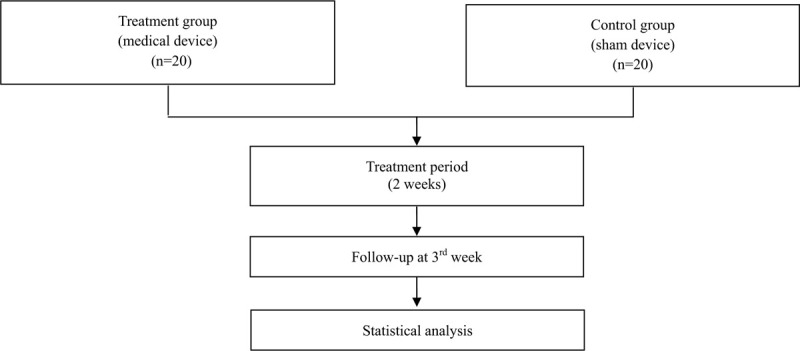
Flowchart of this study.

A total of 40 participants will be recruited for this trial and will be randomly allocated to two parallel groups: the medical device group or the sham device group. The trial will consist of a 2-week intervention period and a 1-week follow-up period. In the intervention period, 10 sessions of treatment will be conducted. The study design is summarized in Figure 1 and Table 1. The study protocol (version 1.2) was developed as required by the Standard Protocol Items: Recommendations for Interventional Trials (SPIRIT) guidelines (Additional file 1).

**Table 1:**
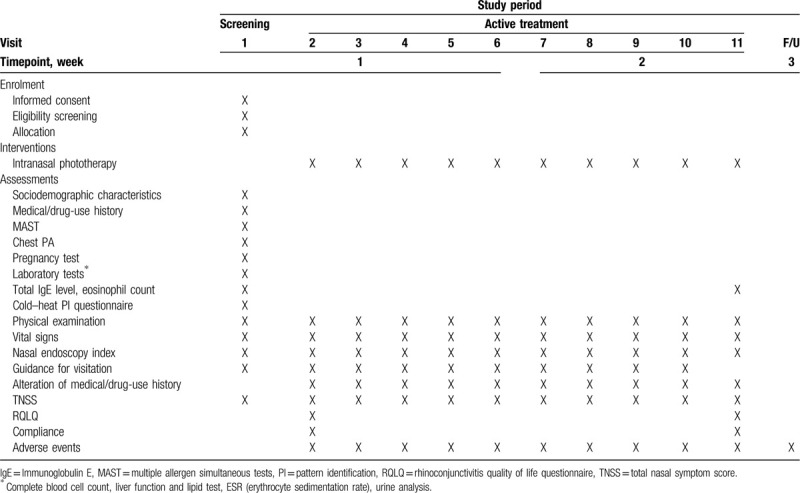
Schedule of enrolment, interventions, and assessments.

### 2.3 Recruitment

The trial will be advertised on the hospital bulletin board, outside the clinics of each center, and the website of the universities, which are located within geographical proximity to the hospital area. The posters will contain brief descriptions and outline the purpose of this study, the inclusion criteria, and the intervention. Written informed consent will be obtained from all study participants before enrolment, and all participants have the right to participate or withdraw from the study at any time without penalty.

### 2.4 Participant inclusion and exclusion criteria

#### 2.4.1 Inclusion criteria

Participants will be included in this study if they meet all of the following criteria:

1. Aged ≥19 years
2. Presence of two or more symptoms of AR (nasal congestion, rhinorrhea, nasal itching, and sneezing)
3. Positive reaction to an allergen in the multiple allergen simultaneous test (MAST)
4. Voluntary participation in this study and signing of the patient consent form after explanation of the purpose of the study.

#### 2.4.2 Exclusion criteria

Participants will be excluded if they meet any of the following conditions:

1. Current use of any of the following medications or inability to stop taking medication that is unsuitable for this clinical trial (however, enrolment in this trial is possible after the suggested minimum wash-out period before Visit 2).
  (a) Use of antihistamines/H1 blockers within the previous week
  (b) Use of corticosteroids (intranasal) within the previous 2 weeks
  (c) Use of corticosteroids (systemic) within the previous 30 days
  (d) Use of anticholinergics within the previous week
  (e) Use of leukotriene receptor antagonists within the previous 4 weeks
  (f) Use of decongestants within the previous 3 days
  (g) Use of tricyclic antidepressants or phenothiazines within the previous 2 weeks
  (h) Use of non-steroidal analgesics within the previous 2 weeks
  (i) Use of other inappropriate medications as judged by the investigator
2. Use of traditional Korean medicine treatments (herbal medicine, acupuncture, moxibustion, cupping, etc) to treat AR within the previous 2 weeks
3. Use of laser therapy to treat AR within the previous 2 weeks
4. Diagnosis of malignant neoplasm, anemia, active pulmonary tuberculosis, infection, or other severe systemic disease
5. Presence of anatomical obstructions or deformities of the nasal cavity or nasal surgery within the previous 6 months
6. Past history of active respiratory disease such as asthma
7. Hypertension or diabetes
8. Treatment with immunotherapy or systemic corticosteroid therapy within the previous 2 years
9. Hypersensitivity reactions to phototherapy or from taking related drugs
10. Presence of a scar on the irradiation spot
11. Pregnant, planning a pregnancy or breast-feeding
12. Participation in other clinical trials within the previous 1 month
13. Difficulty enrolling in this trial or receiving treatment
14. Others: ineligible for participation as judged by the investigator

### 2.5 Intervention

Participants will be randomly assigned to two groups: an experimental group (medical device that emits low-level laser light) and a control group (sham device that emits LED light) at a ratio of 1:1. Participants will receive phototherapy in each nasal cavity for 20 min, 5 times a week for 2 weeks.

The medical device that will be used is the Wellrhino unit (Optowell Co., Ltd., Jeonju-Si, Korea), which emits a low-level laser light consisting of two wavelengths, 670 and 830 nm, with unique sounds; the sham device emits LED light at 650 nm. Both devices will be identical in appearance and characteristics.

Participants will not be allowed to use medications related to AR or therapeutic interventions such as acupuncture and herbal prescriptions for AR during the trial period.

### 2.6 Outcome measures

#### 2.6.1 Primary outcome

The primary outcome is the change in the Total Nasal Symptom Score (TNSS) from baseline to 2 weeks. The TNSS assesses 4 nasal symptoms (nasal congestion, rhinorrhea, nasal itching, and sneezing) on a five-point scale: 0, no symptoms; 1, mild; 2, moderate; 3, severe; and 4, very severe.^[19]^ TNSS will be measured at every visit, and we will analyze the differences in effects between groups.

#### 2.6.2 Secondary outcomes

The secondary outcomes include changes in Rhinoconjunctivitis Quality of Life Questionnaire (RQLQ) score, Nasal Endoscopy Index, total serum IgE level, and eosinophil count level. The RQLQ is an instrument used to assess rhinoconjunctivitis-related QOL and daily activities, which contains 28 items in 7 domains (activity limitation, sleep problems, nose symptoms, eye symptoms, non-nose/eye symptoms, practical problems, and emotional function).^[18,20]^ The RQLQ score will be assessed twice: just prior to the start of the treatment at visit 2 and at the end of the treatment at visit 11.

The Nasal Endoscopy Index assesses nasal cavity conditions according to 4 aspects: color (pale or hyperemia), dryness or dampness, rhinorrhea, atrophy, or edema.^[21]^

The Nasal Endoscopy Index will be evaluated at every visit. The instrument used is the KAZAMA ENT treatment unit (KAU-3000 HARMONY, ENT Co., Ltd., Incheon, Republic of Korea).

We will also examine the total serum IgE level and eosinophil count at screening (visit 1) and visit 11 to evaluate the effects of intranasal phototherapy on allergic and inflammatory reactions.

#### 2.6.3 Other measures

To identify the correlation between the effects of intranasal phototherapy and the cold–heat pattern, we will use the Cold–Heat Pattern Identification Questionnaire. The Cold–Heat Pattern Identification Questionnaire is used to distinguish between cold and heat, which is a characteristic of patients from a Korean traditional medicine perspective. The questionnaire consists of 15 items, which include 8 items related to cold and 7 items related to heat. According to the results of this questionnaire, a patient could be classified as Cold, Hot, neither Cold nor Hot and Cold–Hot complex.^[22]^ The questionnaire will be completed at screening.

### 2.7 Randomization and allocation concealment

Participants who meet all inclusion–exclusion criteria will receive a subject identification code. Accordingly, block randomization will be performed to assign the same number of subjects to the medical device or sham device group. A total of 40 participants will be randomly assigned to each group in a 1:1 ratio. A statistician who is not directly involved in the trial will generate a random allocation sequence using a computer program (Strategic Applications Software [SAS], version 9.3; SAS Institute Inc, Cary, NC), and the allocation sequence will be sealed in a consecutively numbered secure envelope. The allocation for each randomization number will be put in individually seal opaque envelopes. Consent will be obtained by a Korean medicine doctor, and all participants will be informed that they will be assigned to one of two groups. After the participants have signed the informed consent form, the envelopes will be opened.

### 2.8 Blinding

The participants and investigators will be blinded to the group allocation data, which will only be known to the person in charge of the random allocation. The random allocation table will be kept under lock by Medipert, a contract research organization (CRO) not involved in implementing this study. Both medical and sham devices, seemingly the same, will be repackaged and labeled by the device manufacturer (Optowell Co., Ltd., Jeonju-Si, Korea) according to instructions from Medipert before being delivered to the institution (Woosuk Korean Medicine Medical Center). The subjects will receive intranasal phototherapy with their corresponding device, either the medical device or the sham device, according to their randomization number.

### 2.9 Sample size

Since the aim of this study is to determine preliminary feasibility, there will be no sample size calculation. We will conduct a pilot study with 40 participants (20 in the medical device group and 20 in the sham device group) based on the previous pilot study,^[23]^ and we predict that the drop-out rate will be 20%. Thirty-two patients are predicted to complete the study. This sample size is considered to be sufficient to provide power analysis and sample size calculation for subsequent large-scale definitive RCTs.

### 2.10 Data management

The investigators will telephone the participants before every visit to promote participant adherence. If participants fail to attend treatment sessions, we will ask for the reasons for the absence and encourage visits through telephone contact.

Regular monitoring will be conducted to control the quality of the data according to the planned protocol and standard operating procedures. Medipert, the CRO of this study, will send a clinical research associate (CRA) to Woosuk Korean Medicine Medical Center every week. The CRA will check whether the trial is being conducted according to the approved protocol and whether the data are adequately recorded. Each participant's contact or identifying information will be separately stored. Personal identity will not be disclosed. Additionally, investigators will not collect any other personal data. All data and records related to the clinical trials will be kept in a locked cabinet. Documents related to this clinical trial should be kept for 10 years after completion of the clinical trial under the supervision of the custodian after completion of the clinical trial result report.

### 2.11 Safety and adverse events

The participants will be asked to voluntarily report information about adverse events (AEs), and the investigator will assess any adverse events and vital signs at every visit to monitor the participant's compliance and safety. AEs such as nasal dryness, nasal septum perforation, burning, epistaxis (nosebleed), inflammation or stinging, or other AEs reported during the study period will be recorded on the adverse events form in accordance with the guidelines of the Institutional Review Board. The symptoms, date of occurrence and disappearance, severity, causal relationship with the treatment, other medications or treatments suspected to have caused the AE, and treatment for the AE will be recorded in detail. When severe AEs occur, the investigator will report the event to the Institutional Review Board (IRB) immediately and decide whether to terminate the study. Additionally, adverse events associated with the intervention during the trial will be appropriately handled, and we will provide corresponding compensation according to the insurance policy.

### 2.12 Statistical analysis

The statistical analysis will be performed using SAS by an independent statistician who is blinded to the randomized allocation of participants.

All analyses will follow the full analysis set based on the principle of intention-to-treat (ITT); the per-protocol (PP) analysis set will be used for the sensitivity analysis. We will apply the last-observation-carried-forward (LOCF) rule for missing data.

The demographic characteristics and data of both groups will be summarized by means and standard deviations for the continuous variables, and frequencies and percentages will be presented for categorical variables. Differences in baseline between groups will be evaluated by using independent t tests or Mann–Whitney *U* tests for continuous variables and the chi-squared test or Fisher exact test for categorical variables.

Two-sample *t* tests or Wilcoxon rank-sum tests will be used to analyze differences in the primary and secondary outcomes before and after treatment in each group. To demonstrate superiority, we will check whether the upper limit of the one-sided 95% confidence interval (CI) of the difference in the primary outcome between the 2 groups is less than the superiority margin (0.28 points).^[24]^

All participants who participated in at least 1 session will undergo an adverse effect evaluation. All adverse effects reported during the clinical trial will be analyzed. To compare groups and the incidence of adverse events related to the two groups, the chi-square test or Fisher's exact test will be used. The level of significance will be set at 0.05 (2-tailed), except in the case of the primary outcome.

No interim analysis will be performed due to the short duration of the trial.

### 2.13 Ethics

The study will be conducted in compliance with the Declaration of Helsinki and the Korean Good Clinical Practice (KGCP) guidelines. This study protocol was approved by the Institutional Review Board of the Woosuk University Korean Medicine Hospital (WSOH IRB M1807–01–02) and registered in the Clinical Research Information Service (CRIS), which is a primary registry of the World Health Organization International Clinical Trials Registry (https://cris.nih.go.kr/cris/en/search/search_result_st01.jsp?seq=16766). Protocol modifications will be provided to the Institutional Review Board and the trial registry for their approval. Prior to participation, participants will be fully informed about the study and voluntarily sign an informed consent form.

The results of the study will be shared with health care professionals, traditional medicine associations, and other relevant organizations through conferences and will be published in peer-reviewed journals.

## 3 Discussion

Due to the limitations of conventional therapies, there is a need to develop alternative treatments for AR. Among them, intranasal phototherapy has received growing interest as an alternative choice to relieve the symptoms of AR patients who cannot take conventional medications because of contraindications or who do not respond well to conventional therapy. Therefore, to investigate the effects and safety of intranasal phototherapy for AR, this proposed trial has been rigorously designed as a randomized, double-blind, placebo-controlled, clinical trial to minimize risks of bias.

In this study, participants will be blinded during the intervention processes using a sham device that is the same in appearance as the treatment device. As the devices will be labeled by the device manufacturer who is not involved in this trial, the investigator operating the device will be blinded to reduce performance bias.

This RCT adopts objective measures such as serum IgE and eosinophil count as well as questionnaires such as the RQLQ to further investigate the mechanisms of action of intranasal phototherapy for AR. In addition, we will utilize the last-observation carried forward (LOCF) rule and intention-to-treat (ITT) approach to manage the attrition bias.

There are some limitations related to the small sample size and lack of sample size calculations. Additionally, we did not stratify demographic factors and characteristics of AR, such as severity and duration of AR, in the randomization process. Therefore, based on the findings of this study, subsequent large-scale definitive RCTs will be needed to confirm the effects and safety of intranasal phototherapy for the treatment of AR.

This protocol was approved by the Korea Food and Drug Administration (KFDA) Investigational Medical Device Application (IMDA). We expect that the results of this study will provide preliminary evidence for broadening the approval range of phototherapy for use in “allergic rhinitis.” Additionally, the results of this study will be the basis for the evaluation of the New Health Technology Assessment (new-HTA).

In 2016, the Korean Medicine Clinical Practice Guideline (CPG) was developed to establish evidence for Korean medicine and strengthen health insurance coverage, and AR is one of the target diseases of the CPG. We expect that the results of this study will contribute to the development of treatment interventions in Korean medicine and establish evidence for “intranasal phototherapy,” which is included as a key question in the Allergic Rhinitis Korean Medicine Clinical Practice Guideline.

## 4 Conclusion

This pilot RCT is designed as the first step to explore intranasal phototherapy for AR. The results of this study are expected to be the basis for the design and implementation of large-scale RCTs investigating the effects and safety of intranasal phototherapy for AR.

## Author contributions

**Conceptualization:** Donghyo Lee.

**Data curation:** Jeonghun Kim, Jeongin Kang.

**Funding acquisition:** Donghyo Lee.

**Investigation:** Jeongin Kang, Jeonghun Kim, Donghyo Lee.

**Methodology:** Jeongin Kang, Goeun Lee, Youngeun Kim, Sunju Park, Donghyo Lee.

**Writing – original draft:** Jeongin Kang, Goeun Lee.

**Writing – review & editing:** Sunju Park, Donghyo Lee.
